# Supplementary material for: The interactome of intact mitochondria by cross-linking mass spectrometry provides evidence for coexisting respiratory supercomplexes
Source: Mol Cell Proteomics. 2017 Dec 8;17(2):216–32. doi: 10.1074/mcp.RA117.000470 (PMC5795388; doi:10.1074/mcp.RA117.000470)
Supplement: Supplemental Data [file supp_RA117.000470_133922_0_supp_23978_fzffwf.zip › spectra_annotation/mito_DR_spectra_annotation/266-1-4-1-5-1.pdf]

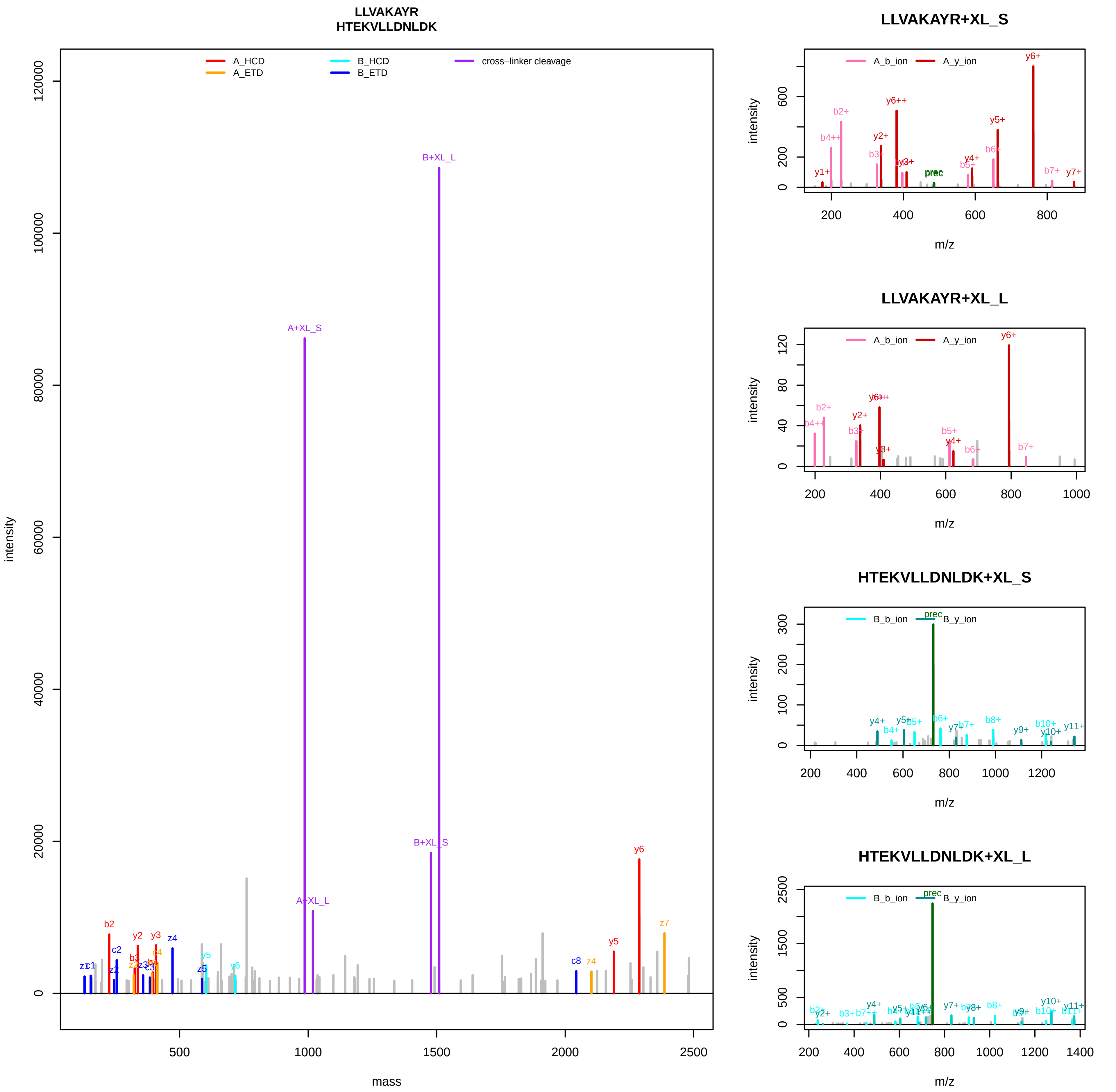
LLVAKAYR
HTEKVLLDNLDK
A_HCD
A_ETD
B_HCD
B_ETD
cross−linker cleavage
B+XL_L
A+XL_S
B+XL_S
A+XL_L
b2
y2
y3
z4
c2
c8
z4
y5
y6
z7
y5
y6
z5
intensity
mass
0
20000
40000
60000
80000
100000
120000
500
1000
1500
2000
2500
LLVAKAYR+XL_S
A_b_ion
A_y_ion
y6+
y6++
b2+
b4++
y2+
y5+
y4+
y7+
y1+
b7+
prec
intensity
m/z
LLVAKAYR+XL_L
A_b_ion
A_y_ion
y6+
y6++
b2+
y2+
b5+
y4+
b7+
intensity
m/z
HTEKVLLDNLDK+XL_S
B_b_ion
B_y_ion
prec
y4+
b4+
y5+
b6+
b7+
b8+
y9+
b10+
y10+
y11+
intensity
m/z
HTEKVLLDNLDK+XL_L
B_b_ion
B_y_ion
prec
y2+
y4+
y7+
y8+
b8+
y10+
y11+
intensity
m/z
